# Retrospective Review of Ocular Point-of-Care Ultrasound for Detection of Retinal Detachment

**DOI:** 10.5811/westjem.2015.12.28711

**Published:** 2016-03-02

**Authors:** Bradley Jacobsen, Sari Lahham, Shadi Lahham, Amy Patel, Sophia Spann, John C. Fox

**Affiliations:** * University of California Irvine School of Medicine, Irvine, California; † University of California Irvine Medical Center, Department of Emergency Medicine, Orange, California; ‡ University of California Irvine, Gavin Herbert Eye Institute, Department of Ophthalmology, Irvine, California; § University of California Irvine, Department of Emergency Medicine, Orange, California

## Abstract

**Introduction:**

Retinal detachment is an ocular emergency that commonly presents to the emergency department (ED). Ophthalmologists are able to accurately make this diagnosis with a dilated fundoscopic exam, scleral depression or ophthalmic ultrasound when a view to the retina is obstructed. Emergency physicians (EPs) are not trained to examine the peripheral retina, and thus ophthalmic ultrasound can be used to aid in diagnosis. We assessed the accuracy of ocular point-of-care ultrasound (POCUS) in diagnosing retinal detachment.

**Methods:**

We retrospectively reviewed charts of ED patients with suspected retinal detachment who underwent ocular POCUS between July 2012 and May 2015. Charts were reviewed for patients presenting to the ED with ocular complaints and clinical concern for retinal detachment. We compared ocular POCUS performed by EPs against the criterion reference of the consulting ophthalmologist’s diagnosis.

**Results:**

We enrolled a total of 109 patients. Of the 34 patients diagnosed with retinal detachment by the ophthalmologists, 31 were correctly identified as having retinal detachment by the EP using ocular POCUS. Of the 75 patients who did not have retinal detachment, 72 were ruled out by ocular POCUS by the EP. This resulted in a POCUS sensitivity of 91% (95% CI [76–98]) and specificity of 96% (95% CI [89–99]).

**Conclusion:**

This retrospective study suggests that ocular POCUS performed by EPs can aid in the diagnosis of retinal detachment in ED.

## INTRODUCTION

Ocular complaints represent between 2–3% of emergency departments (ED) visits. This includes many vision-threatening diagnoses such as retinal detachment, occurring in 3–4% of patients presenting with ocular complaints.^1^–^3^ Retinal detachment is one of the few ophthalmologic emergencies commonly seen in the ED.^4^ It requires immediate assessment, diagnosis and treatment to prevent permanent vision loss.^5^

Diagnosis of suspected retinal detachment often entails assessing visual acuity with the Snellen chart, confrontational visual field testing, slit lamp biomicroscopy and direct ophthalmoscopy after pharmacological dilation.^6^ In a fast-paced ED setting, these tests can be time consuming and require proficiency with ophthalmological tools. Over the past decade, ophthalmology-specific courses and formal ophthalmology rotations have declined significantly across medical schools within the United States. This puts emergency physicians (EPs) and the patients they serve in a compromising position to correctly diagnose and/or properly refer their patients.^7^ Additionally, in rural settings, an on-call ophthalmologist may not be available. The use of bedside ocular point-of-care ultrasound (POCUS) has the potential to properly identify ocular emergencies in these settings.

Ophthalmic ultrasound has become useful in diagnosing various ocular pathologies. Originally, ocular ultrasound was used less frequently due to low-resolution ultrasound machines; however, newer machines have improved diagnostic capability.^8^,^9^ A study by Blaivas’ et al. found that ocular POCUS could be used in the diagnosis of various ocular pathologies including retinal detachment, central retinal artery occlusion, lens dislocation vitreous hemorrhage and vitreous detachment.^10^ To date there have been two prospective studies that specifically look at the use of ocular POCUS to diagnose retinal detachments in the ED. Yoonessi et al prospectively studied 48 patients and found a sensitivity and specificity of 100% and 83% respectively.^11^ Similarly Shinar et al prospectively studied 90 patients and found a sensitivity and specificity of 97% and 92% respectively.^12^ These studies had various limitations including small sample sizes and large confidence intervals.

Our goal was to retrospectively investigate the outcomes of patients who had an ocular POCUS in the ED for suspected retinal detachment. We compared this diagnosis to the criterion reference diagnosis of the consulting ophthalmologist. We aimed to expand on previous research by studying a larger patient population.

## METHODS

### Study Design

After institutional review board approval, we conducted a retrospective chart review of patients who were billed for an ocular POCUS between the dates of July 2012 and May 2015. Medical record numbers were then used to identify patients who had both ocular POCUS and formal ophthalmologic consultation. Research personal recorded these data, using a systematic approach on a standardized data collection sheet.^13^ Collected data included patient age, gender, basic demographics, POCUS diagnosis and ophthalmology diagnosis. Reviewers were blinded to patient ultrasound findings. A second reviewer confirmed all documented findings for agreement. We excluded patients if there was any concern for globe injury, or if they did not get a formal ophthalmologic consultation in the ED. The EP diagnosis, after performing the ocular POCUS, was compared to the ophthalmologist clinical diagnosis to determine the sensitivity, specificity, positive predictive value (PPV) and negative predictive value (NPV) of ocular POCUS. At our institution EPs perform ocular POCUS prior to evaluation by Ophthalmology. This ensured that EPs diagnosis was not influenced by an ophthalmology exam.

It should be noted that the emergency medicine (EM) ocular standard of care includes assessments of visual acuity, pupil size, and performance of direct ophthalmoscopy as well as slit lamp examinations. All were attempted prior to obtaining ocular POCUS.

### Study Setting

**Figure 1:**
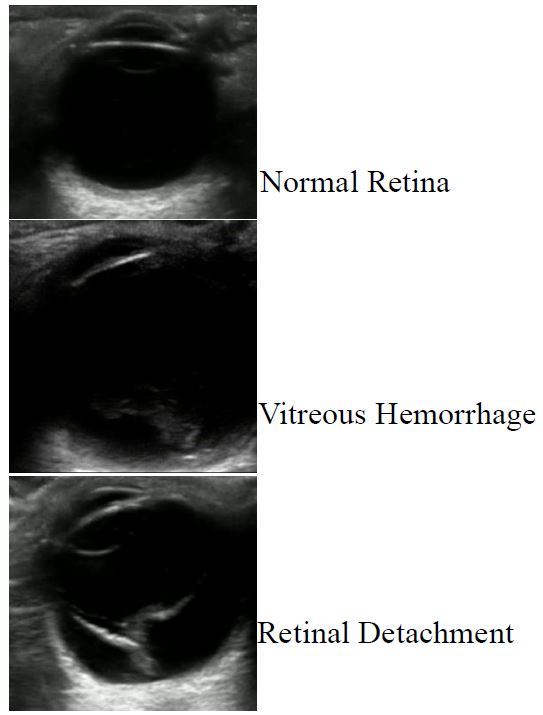
Ocular ultrasound representing normal retina, vitreous hemorrhage and retinal detachment.

The study took place at an urban university hospital ED that supports both EM and ophthalmology residency-training programs, as well as an emergency ultrasound fellowship. The annual ED census is approximately 48,000 patient visits, and 24-hour ophthalmologic consultation is available. A total of 26 different EPs evaluated patients presenting with symptoms concerning for retinal detachment. These physicians were a mixture of resident and attending physicians who have training in various applications in POCUS. This included a 30-minute lecture followed by supervised hands-on scanning of three volunteer models. The training introduced the physicians to ocular POCUS and highlighted the differences between normal retina, retinal detachment and vitreous hemorrhage (Figure 1). No additional training was provided to ED attending or resident physicians.

### Study protocol

After the EP ocular physical examination described above, patients underwent ocular POCUS performed by the EP when the etiology could not be determined by physical exam. This consisted of using Sonosite M-Turbo ultrasound machine with a 7.5MHz, high frequency, linear probe (Sonosite, Botthell, WA). Both sagittal and transverse views of the affected eye were obtained. The posterior chamber on both sides of the optic nerve was examined for presence or absence of a detached retina. The results of the ocular POCUS were obtained from the electronic medical record. Ophthalmologic consultation was obtained following POCUS. The ophthalmologist’s exam, performed by a combination of residents and attendings, included visual acuity, pupils, slit lamp exam, dilation, and indirect ophthalmoscopy with scleral depression. If the ophthalmologist had a poor posterior view and inconclusive diagnosis with the aforementioned examination skills, a B-scan ultrasound was performed.

### Statistical Analysis

We calculated sensitivity, specificity, positive and NPVs with 95% confidence intervals for POCUS compared with formal ophthalmologic consulting physician’s diagnosis. These parameters were calculated in the usual manner with confidence intervals by the efficient-score method with continuity correction.

## RESULTS

A total of 142 patients who underwent ocular POCUS between July 2012 and May 2015 were identified. Of these patients, 109 received ophthalmology consultation following EP-administered ocular POCUS and were included in our study. These patients ranged in age from 8 to 84, with a mean of 49.3 years (+/−16.1). Thirty-five of these patients presented with loss of vision (18 with progressive vision loss and 17 with sudden vision loss), 23 with floaters, flashes and/or spots, 10 with ocular irritation and 41 with blurry vision and/or decreased vision. There were 52 males and 57 females enrolled in the study. Twenty-six different EM attending physicians and 30 EM resident physicians used ocular POCUS in these 109 cases. Each attending physician accounted for between 1–30 enrolled patients, with a median of four scans.

**Figure 2:**
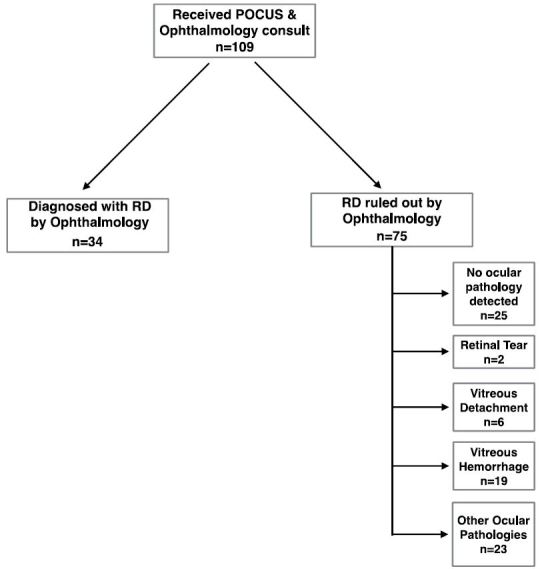
Algorithm for retinal detachment diagnosis using ultrasound. *POCUS*, point of care ultrasound; *RD*, retinal detachment

A total of 34 patients (31%) were diagnosed with some form of retinal detachment by the ophthalmologic consulting physician (Figure 2). POCUS demonstrated the ability to detect 31 of these patients as having retinal detachment, resulting in a sensitivity of 91% (95% CI [76–98]). POCUS correctly ruled out retinal detachment in 72 of the 75 cases determined by ophthalmology to be negative for retinal detachment, resulting in a specificity of 96% (95% CI [89–99]). Thus, the PPV observed was 91% (95% CI [76–98]) while the NPV observed was 96% (95% CI [89–99], Table). Of the 75 negative cases, 19 were diagnosed with vitreous hemorrhage, six were diagnosed with vitreous detachment, 25 were determined to have no ocular pathology, two were diagnosed with retinal tear and 23 were diagnosed with other ocular pathologies (lens dislocation, increased intracranial pressure, pre-retinal heme, branch retinal vein occlusion etc.) (Figure 2).

## DISCUSSION

To date, this is the largest retrospective study performed investigating the use of ocular POCUS to diagnose retinal detachment. Our findings validate the findings of previous studies and demonstrate that EPs can use ocular POCUS to identify retinal detachments. Prompt and accurate diagnosis by an EP can lead to better communication with the ophthalmology consultant and improve quality of care.

An ophthalmologist has an advanced skill set, including a dilated fundoscopic exam with or without scleral depression and a specialized ophthalmic ultrasound machine to accurately differentiate retinal detachment from a retinal tear.^14^ EPs trained in the performance and interpretation of ophthalmic ultrasound may be better able to transition care to an ophthalmologist. The use of ophthalmic ultrasound as a diagnostic modality is not meant to replace the role of the ophthalmologist, but rather serve as an adjunct to improve quality of eye care in the ED. This plays an especially important role in rural communities where an ophthalmologist may not be readily accessible.^15^–^18^

In a recent study done by Esparaz et al, medical students were evaluated on their preparedness in managing and diagnosing common ocular pathology and found that both second-year and fourth-year medical school students, on average, did not pass the ophthalmology proficiency quiz.^7^,^19^ They discuss the decline in ophthalmologic clinical experience within medical schools and further suggest that residents may not have the appropriate training to properly manage ophthalmologic conditions.^7^,^19^ With ultrasound being incorporated to a greater degree in medical education at all levels, POCUS becomes a more reasonable and readily available tool to be used in the ED, and can help bridge the growing gap in EP ophthalmologic examination skills.^20^,^21^ However, ultrasound training is not uniform across medical schools, residency programs, or teaching hospitals across the country.^22^ Davis, et al showed that sonologists with highest confidence in their POCUS skills had a much higher accuracy of emergency ultrasound diagnosis.^23^ Thus, it is critical that those using POCUS in emergent situations are trained to a proper level to avoid making critical mistakes in diagnosis.^24^,^25^

At this time, it is unclear what degree of sonographic training is required for EPs to diagnose retinal detachment. Currently, no metric or level of training can be measured to satisfy a minimum level.^26^ Blaivas et al show a sensitivity of 100% and specificity of 100% for EPs to diagnose retinal detachment using POCUS.^10^ Given that ultrasound is operator dependent, this may not be uniform to all EPs. In our study, resident physicians in different years of training performed and interpreted ocular POCUS in the presence of an EM attending physician. Future studies will need to assess the number of ocular POCUS that a practitioner would need to perform to be deemed competent to independently diagnose retinal detachment.

## LIMITATIONS

There is currently no standardized protocol for diagnosing retinal detachments using ocular POCUS. Thus, results may have varied depending on ultrasound experience. A larger patient population is needed to confirm the accuracy of ocular POCUS in diagnosing retinal detachments. One attending physician accounted for nearly a third of enrolled patients. This may have introduced bias potentially inflating the value of POCUS for retinal detachment. Finally, since this was a retrospective study, EPs were not blinded to any past medical history and/or past surgical history, which may have influenced the ultrasound interpretation. Similarly, it was possible that ophthalmologists may have been able to access the ocular POCUS results prior to performing their own consultation. Our study results have shown that 31% of enrolled patients were diagnosed with retinal detachment. This may be due to the fact that our practitioners documented POCUS on patients with high clinical suspicion for retinal detachment. Despite these limitations, we feel that EP ocular POCUS is a viable option with test characteristics that may aid in the diagnosis and reduce the time to treatment for patients with retinal detachments in the ED setting.

## CONCLUSION

Ultimately, the use of ocular ultrasound to detect retinal detachment has important implications in the ED patient course. Our data support the findings of previous smaller-scale prospective and retrospective studies. Further large-scale prospective trials must be conducted to determine the training required for EPs to correctly diagnose retinal detachment via POCUS. With further validation, ocular POCUS has the potential to differentiate patients who need emergent ophthalmologic consultation from those who can follow up in the outpatient setting.

## Figures and Tables

**Table:** Point-of-care ultrasound (POCUS) findings compared with ophthalmology diagnosis.

	RD	No RD	Total
POCUS			
RD	31	3	34
No RD	3	72	75
Total	34	75	109
Sensitivity			91% (31/34); 95% CI [76–98]
Specificity			96% (72/75); 95% CI [89–99]
PPV			91% (31/34); 95% CI [76–98]
NPV			96% (72/75); 95% CI [89–99]

*POCUS,* point-of-care ultrasound; *RD,* retinal detachment; *PPV*, positive predictive value; *NPV*, negative predictive value
